# Immunohistochemical characteristics of thyroid-like low-grade nasopharyngeal papillary adenocarcinoma: A case report and review

**DOI:** 10.1097/MD.0000000000032655

**Published:** 2023-01-27

**Authors:** Hongjie Guo, Zixiong Zhang, Chuying Huang, Jiao Qiu, Xiaochun Qu

**Affiliations:** a Graduate school of Hubei University of Medicine, Hubei, China; b Department of Otolaryngology Head and Neck Surgery, The Central Hospital of Enshi Tujia and Miao Autonomous Prefecture, Enshi Prefecture, China.

**Keywords:** diagnosis, immunohistochemistry, low-grade papillary adenocarcinoma, nasopharynx

## Abstract

Thyroid-like low-grade nasopharyngeal papillary adenocarcinoma (TL-LGNPPA) is a rare nasopharyngeal malignant tumor that is easy to misdiagnose. Immunohistochemistry plays an indispensable role in distinguishing TL-LGNPPA from other malignancies. However, there is no article to summarize the immunohistochemical characteristics of TL-LGNPPA. Herein, we report a case of TL-LGNPPA and present the immunohistochemical results reported in the Chinese literature.

**Methods:**

An electronic search of the CNKI (China National Knowledge Infrastructure) database was performed. From our literature survey, 53 cases of TL-LGNPPA (including the case described in this report) have been identified in China. We summarize the Chinese literature's clinical characteristics, immunohistochemical results, treatments, and prognosis of 53 cases.

**Results:**

Based on our literature survey, 53 cases of TL-LGNPPA (including the case described in this report) have been reported in China. We found TL-LGNPPA and papillary thyroid carcinoma were positive for TTF-1 and CK19. TL-LGNPPA was negative for TG and PAX-8, whereas papillary thyroid carcinoma was positive for TG and PAX-8. However, negative expression of TTF-1 and positive expression of TG were also found in some TL-LGNPPA cases. Our literature survey found that all TL-LGNPPA cases were negative for PAX-8.

Therefore, we suggest that simultaneous immunohistochemical determination of TTF-1 and CK19, as well as TG and PAX-8, can increase the diagnostic accuracy of TL-LGNPPA.

**Conclusion:**

The 4th edition of the World Health Organization Classification of Head and Neck Tumors (WHO-HNT) indicates that NPPA with positive expression of cytokeratin 19 (CK19) and TTF-1 and negative expression of TG is called TL-LGNPPA.

## 1. Introduction

Thyroid-like low-grade nasopharyngeal papillary adenocarcinoma (TL-LGNPPA) is a rare disease that originates from the nasopharyngeal epithelium, accounting for 0.38 and 0.48% of all malignant neoplasms in the nasopharynx.^[1]^ It was first reported by Wenig et al^[2]^ in 1988. To our knowledge, fewer than 100 cases of TL-LGNPPA have been reported in the literature, 53 of which (including the case described in this report) have been identified in China. Herein, we summarize the clinical characteristics, immunohistochemical results, treatments, and prognosis of the 53 cases from the Chinese literature and suggest recommendations for diagnosis.

## 2. Case report

**Figure 1.**
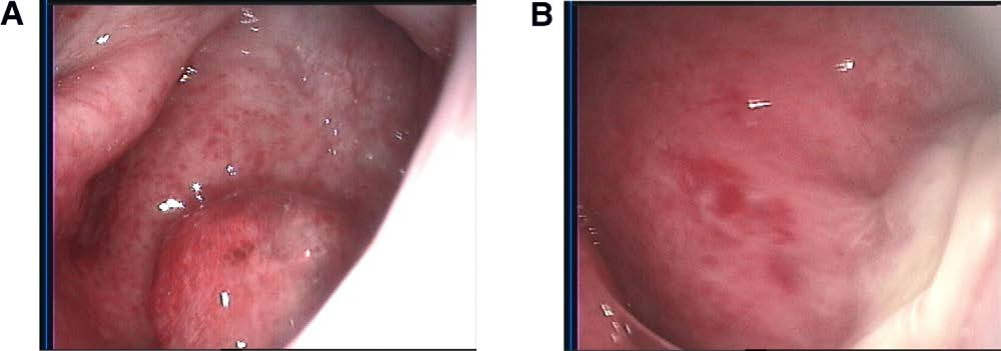
(A) Nasal endoscopy identified a pedunculated polypoid mass with a smooth surface. (B) Pseudomembrane formation of the wound on the second day after plasma knife surgery.

**Figure 2.**
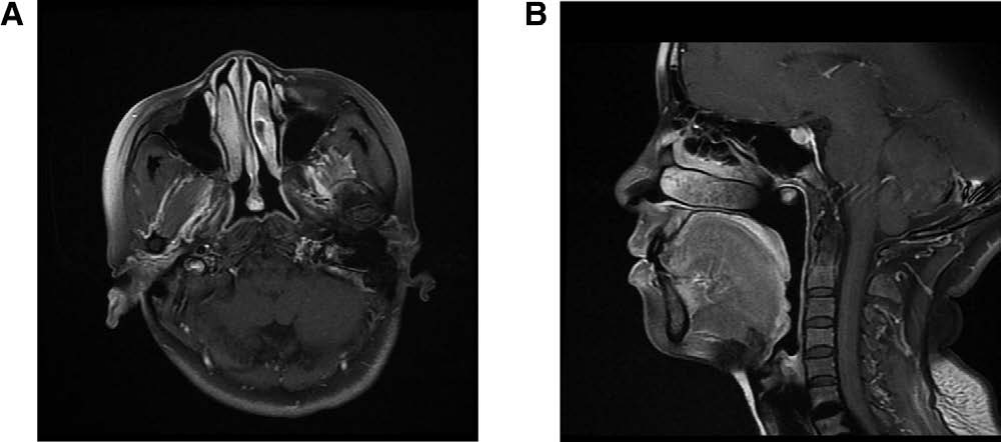
(A) MRI showed a 0.7 × 0.9 × 0.9 cm sized, heterogeneously contrasted mass arising from the posterior edge of the nasal septum. (B) MRI showed a 0.7 × 0.9 × 0.9 cm sized, heterogeneously contrasted mass located in the posterior wall of the parietal nasopharynx.

The case is of a 47-year-old Chinese woman with a complaint of rhinorrhea accompanied by bleeding repeatedly for 3 months. Nasal endoscopy identified a pedunculated polypoid mass with a smooth surface that measured approximately 1.0 × 0.5 cm arising from the posterior edge of the nasal septum (Fig. 1A). Computed tomography revealed a nasopharyngeal tumor 0.7 × 0.9 × 0.9 cm in size with no signs of local infiltration and bone erosion. Magnetic resonance imaging showed a 0.7 × 0.9 × 0.9 cm sized, heterogeneously contrasted mass arising from the posterior edge of the nasal septum (Fig. 2A) and located in the posterior wall of the parietal nasopharynx (Fig. 2B). Ultrasonographically, there were no pathological lymph nodes, and the thyroid gland was normal. The thyroid function tests were unremarkable. The patient received endoscopic nasopharyngeal total mass excision (plasma knife surgery).

**Figure 3.**
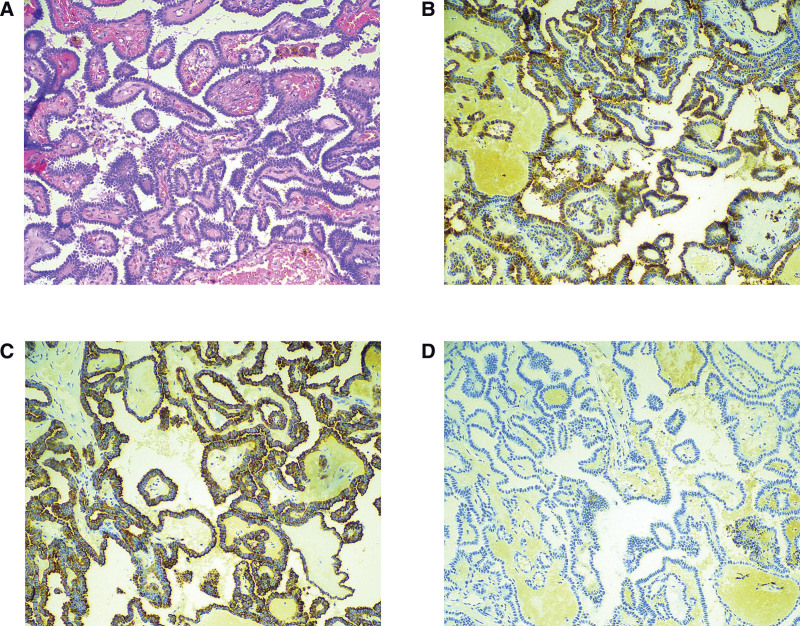
(A) Tumor cells showed a papillary configuration with hyalinized fibrovascular cores, similar to thyroid papillary carcinoma (H&E staining, ×100). (B) Immunohistochemical staining shows that Galectin-3 is overexpressed (immunohistochemistry, ×100). (C) Tumor cells are strongly positive for CK19 (immunohistochemistry, ×100). (D) Tumor cells are negative for TG (immunohistochemistry, ×100).

Histological examination of the biopsy specimen showed that the nasopharyngeal neoplastic tissue consisted of a papillary configuration with hyalinized fibrovascular cores, similar to thyroid papillary carcinoma (Fig. 3A). The papillae were complex and tightly packed, and most were lined with cuboidal or columnar epithelia.

Immunohistochemical studies (Fig. 3B–D) showed that the neoplastic cells were positive for CK19, CK7, TTF-1, Galectin-3, and HBME-1. There was no immunoreactivity for CK5/6, PAX-8, S-100, SOX-10, GATA-3, BRAF-V600E, TPO, or TG. Negative results were revealed by in situ hybridization investigations of Epstein–Barr virus. In the most concentrated area, the Ki-67-labeling index reached 1%. According to these findings, the patient was diagnosed with TL-LGNPPA. No adjuvant chemotherapy or radiotherapy was performed after surgery. Pseudomembrane formation of the wound on the second day after plasma knife surgery (Fig. 1B), and follow-up data showed no signs of local recurrence up to 6 months after complete surgical removal.

## 3. Discussion

Our literature survey revealed that the majority of TL-LGNPPA cases reported in the literature are from China and have been described in the Chinese language. An electronic search of the CNKI (China National Knowledge Infrastructure) database was performed using the following keywords: thyroid, nasopharyngeal, and adenocarcinoma. Fifty-three case reports of patients with TL-LGNPPA were identified.

Of the 53 cases from China, 29 were male patients, and 24 were female patients. The male-female ratio was 1.21:1. The median age was 38 (range, 16 to 69) years. The most common clinical manifestations were nasal obstruction (46.2%), epistaxis and rhinorrhoea with blood (34.6%), and foreign body sensation of the nasal cavity, nasopharynx, and pharynx (36.5%). Only 7.5% of patients experienced headache, dizziness, and hoarseness, and 3.8% were discovered through physical examination. Most tumors originated from the posterior roof of the nasopharynx and the posterior edge of the nasal septum (90.9%), and a few tumors were located in the lateral wall of the nasopharynx (8.8%). There was one case located in the sphenoid sinus, showing invasion into the sphenoid bone and skull base clivus bone. The tumor size ranged from 0.2 to 3.5 cm. Most of the tumors were polypoid masses, 37.2% of which were pedicled. The treatments varied: 50 patients received surgical treatment; only one patient received postoperative radiotherapy; 5 patients received preoperative biopsy; The treatment methods of 3 patients were not specified. No other adjuvant treatment strategies were found in any of the literature. The median follow-up was 12 months (range, 1–116 months), and 58% were followed for more than 1 year. Only one patient experienced recurrence 14 months after surgery, and 52 patients had no locoregional recurrence or distant metastases.

Microscopically, papillary adenocarcinoma with positive cytokeratin staining indicated that the papillary adenocarcinoma originated from the surface epithelium of the nasopharyngeal mucosa rather than from submucosal seromucous glands. Immunohistochemical features and imaging data suggested that the relationship between TL-LGNPPA and the thyroid is unclear. Most nasopharyngeal tumors originating from the nasopharyngeal epithelium are often associated with Epstein–Barr virus. However, based on our literature survey, 36 patients were negative for EBER, indicating that the cases of the disease in these patients were not related to EBV. Wu et al^[3]^ used the PCR method to detect HPV in tumor cells, and the results were negative. Zhang et al^[4]^ labeled HPV-16/18-E6 and P16 with immunohistochemistry, and high-risk HPV-DNA was not detected, suggesting that tumorigenesis is not related to high-risk HPV infection. Radiation exposure is a risk factor for the development of papillary thyroid carcinoma (PTC). Kakkar et al^[5]^ reported the case of a patient diagnosed with TL-LGNPPA after brain tumor radiation for 5 years, and Zhou et al^[6]^ reported the case of a patient who had TL-LGNPPA with bilateral PTC. Therefore, radiation exposure could be a shared pathogenetic factor between these 2 histologically similar tumors. Wang et al^[1]^ found that most of the patients were Asian (76.1%). At present, no clear risk factors for the occurrence of TL-LGNPPA have been found.

It is important to distinguish TL-LGNPPA from nasopharyngeal metastatic PTC due to their similar pathological characteristics and similar expression of TTF-1. Immunohistochemistry, therefore, plays an indispensable role in distinguishing TL-LGNPPA from other malignancies. Herein, we present the immunohistochemical results reported in the Chinese literature (Table 1).^[4,6–31]^

**Table 1:** Immunohistochemical results from the 53 reviewed case reports.

Immunological marker	Number of cases studied	Positive	Positive rate (%)
TTF-1	48	46	95.8
CK7	38	38	100.0
CK19	29	29	100.0
PAX-8	16	0	0.0
TG	46	0	0.0
BRAF-V600E mutation	24	1	4.2
Vimentin	37	34	91.9
EMA	28	28	100.0
CD117	16	12	75.0
CK (pan)	28	28	100.0
S-100	39	2	5.1
CK20	31	0	0.0
CK5/6	26	1	3.8
CD15	11	0	0.0
NapsinA	18	0	0.0
CDX-2	15	0	0.0
SMA	19	0	0.0
HPV-16/18-E6	6	0	0.0
CEA	10	0	0.0
GFAP	12	0	0.0
P63	26	3	11.5
P40	5	0	0.0
TPO	4	0	0.0
EBER	36	0	0.0

Previous studies have indicated that TTF-1 positivity is the most characteristic immunohistochemical feature of TL-LGNPPA. Lai et al^[32]^ reported TTF-1 expression in all 14 cases. Our literature survey found TTF-1 expression in 46 of 48 cases reported in the Chinese literature, and 27 of 46 cases showed diffuse strong positive expression. TTF-1 is a thyroid transcription factor involved in the development of the embryonic thyroid, which is coded by NKX2-1, which shows diffuse strong positive expression in thyroid cancer and lung cancer.^[33–35]^ Oishi et al^[35]^ suggested 3 mechanisms to explain the abnormal TTF-1 expression in TL-LGNPPA: TL-LGNPPA may originate from ectopic thyroid tissue. Gene rearrangement affecting TTF-1/NKX2-1 may cause the aberrant expression of TTF-1. Genetic instability and the reprogramming of cancer cells may cause dedifferentiation and result in deregulation of TTF-1/NKX2-1. However, few cases have been reported at present, and these presumptions are poorly supported.

In addition to TTF-1, another well-known thyroid-specific transcription factor is Pax-8. It is related to thyroid differentiation and is expressed in normal thyroid follicular epithelium, tumor thyroid follicular epithelium, kidneys, and Müllerian organs.^[36]^ Ozcan et al^[36]^ conducted a comprehensive immunohistochemical study of PAX8 and found that all 65 follicular and papillary thyroid neoplasms demonstrated positive staining for PAX-8 in virtually 100% of tumor cells. From our literature survey, 16 cases did not express PAX-8. Therefore, PAX-8 is a helpful marker to distinguish TL-LGNPPA from metastatic PTC.

TG is a secretory protein produced by vertebrates that is synthesized in thyroid cells and stains positively in PTC.^[1]^ In this literature review, all 46 patients had negative TG staining. Zhang et al^[37]^ identified 16 cases, but one reported case of TL-LGNPPA showed focal expression of TG. Therefore, TG immunostaining is the key to distinguishing TL-LGNPPA from PTC, and it is also an important biomarker for the diagnosis of TL-LGNPPA.

Our literature survey also revealed that CK (pan), CK7, CK19, and EMA were overexpressed in TL-LGNPPA, and their positive percentage reached 100%. This confirmed that the tumor cells originated from the covering epithelium of the nasopharyngeal mucosa. On the other hand, the negative expression of S-100, CK5/6, SMA, and P63 revealed that TL-LGNPPA does not originate from the myoepithelium.

Ki-67, which is related to mitosis, is a nuclear antigen expressed in proliferating cells. The Ki-67 proliferation index was 1 to 50% in this survey, and the Ki-67 index was <5% in 90% of cases, suggesting that this type of tumor has a low proliferation ability.

Surgical resection is the first choice for the treatment of TL-LGNPPA, and the surgical approach can be selected according to the size and location of the tumor. Patients with small tumor volumes can undergo complete resection via nasal endoscopy, which causes less trauma and facilitates quick recovery. Lai et al^[32]^ reported that 3 patients received neoadjuvant radiotherapy before the operation and found that the tumor size decreased; the other 2 patients received radiotherapy after the operation. None of the 5 patients had recurrence or metastasis according to the follow-up data. These results indicated that TL-LGNPPA cells are sensitive to radiotherapy. The prognosis of TL-LGNPPA is good. Only one patient with recurrence was reported by Bai et al^[27]^ in the Chinese literature, and no postoperative metastasis has been reported.

## 4. Conclusions

The 4th edition of the World Health Organization Classification of Head and Neck Tumors (WHO-HNT) indicates that NPPA with positive expression of cytokeratin 19 (CK19) and TTF-1 and negative expression of TG is called TL-LGNPPA.^[38]^ In summary, TL-LGNPPA and PTC were positive for TTF-1 and CK19. TL-LGNPPA was negative for TG and PAX-8, whereas PTC was positive for TG and PAX-8. However, negative expression of TTF-1 and positive expression of TG were also found in some TL-LGNPPA cases. Our literature survey found that all TL-LGNPPA cases were negative for PAX-8. Therefore, we suggest that simultaneous immunohistochemical determination of TTF-1 and CK19, as well as TG and PAX-8, can increase the diagnostic accuracy of TL-LGNPPA.

## Acknowledgments

We thank the patient for participating in this study.

## Author contributions

**Data curation:** Hongjie Guo, Jiao Qiu, Xiaochun Qu.

**Writing – original draft:** Hongjie Guo.

**Writing – review and editing:** Zixiong Zhang, Chuying Huang.
